# Development and Application of a Cumate‐Inducible Promoter, P_*gc*_, in *Komagataella pastoris*

**DOI:** 10.1111/1751-7915.70311

**Published:** 2026-02-03

**Authors:** Jiachen Xie, Yazhu Xing, Huiying Luo, Yuan Wang, Wei Zhang, Nan Xu, Bo Liu

**Affiliations:** ^1^ Biotechnology Research Institute Chinese Academy of Agricultural Sciences Beijing China; ^2^ College of Bioscience and Biotechnology Yangzhou University Yangzhou China; ^3^ Institute of Animal Sciences Chinese Academy of Agricultural Sciences Beijing China

**Keywords:** cumate, gene expression, inducible promoter, *Komagataella pastoris*

## Abstract

*Komagataella pastoris* is extensively used as a microbial cell factory for the production of recombinant proteins and high‐value compounds. However, tightly controlled promoter systems responsive to safe and economical inducers are required for precise metabolic and pathway engineering in this yeast species. Cumate‐inducible promoters are an ideal choice due to the safety and low cost of cumate. In this study, we systematically optimised the insertion sites of the CuO operator sequence within the strong promoter P_*GCW14*_ to isolate a high‐activity variant that we designated as P_*GCWCuO03*_. To fine‐tune the expression of the repressor protein CymR, we developed a truncated promoter of P_*GAP*_, designated as P_*GAP200*_. Based on the optimal promoter P_*GCWCuO03*_ and the CymR expression unit, we constructed a robust CymR/CuO‐mediated cumate‐inducible promoter, designated as P_*gc*_, in *K. pastoris*. P_*gc*_ demonstrated outstanding induction properties, resulting in an approximately 11‐fold increase in target protein production following induction. Promoter substitution assays validated the effectiveness of P_*gc*_ in temporal gene expression control, highlighting the significant potential of this promoter for both basic research and industrial bioprocessing applications in synthetic biology and biotechnology in *K. pastoris*.

## Graphical abstract

A novel cumate‐inducible promoter, P_*gc*_, was engineered in *P. pastoris* by optimising the CuO operator and CymR repressor (A). This system achieved an approximately 11‐fold increase in recombinant protein production upon induction (B).

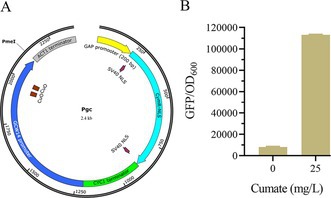

## 1 Introduction

The yeast *Komagataella pastoris* is among the most widely used microbial hosts for recombinant protein production, with more than 4000 heterologous proteins successfully expressed in this host to date (Claes et al. 2024; Vijayakumar and Venkataraman 2024). With recent progress in synthetic biology and metabolic engineering, *K. pastoris* is also increasingly employed as a chassis organism for the synthesis of high‐value bioactive compounds (Gao et al. 2023; Fang et al. 2025). Genetic design is usually necessary to enhance the production efficiency of the target products; this process involves the precise temporal regulation of gene expression using inducible promoters, temperature‐sensitive circuits, or other molecular tools that enable activation or repression of specific metabolic pathways at optimal fermentation stages. Such strategies are employed to maximise product yield, minimise byproduct formation, and improve overall process efficiency.

Promoters are central genetic elements that play critical roles in controlling both the magnitude and timing of gene expression by mediating interactions between RNA polymerase and transcription factors. In *K. pastoris*, several constitutive and inducible promoters have been characterised and adopted for fine‐tuned genetic regulation (Vogl and Glieder 2013; Wu et al. 2023; Liu et al. 2025). Inducible promoters are particularly valuable for controlling the expression of genes encoding toxic proteins or for dynamic metabolic engineering in synthetic biology. The methanol‐inducible promoter P_*AOX1*_ is one of the most widely used due to its strong transcriptional activity and tight regulation (Poodeh et al. 2022). However, methanol is highly flammable and toxic, which limits its suitability for industrial‐scale applications and products with high safety requirements (Duman‐Özdamar and Binay 2021). Alternative promoters have been discovered, including the rhamnose‐inducible promoters P_*LRA3*_ and P_*LRA4*_ (Liu et al. 2016, 2022). Although P_*LRA3*_ shows relatively high activity, the expense of rhamnose as both an inducer and carbon source has hindered its large‐scale use. Other inducible promoters suffer from limited strength, leaky expression, or repression by inexpensive carbon sources such as glucose and glycerol. Therefore, safe, cost‐effective inducible promoters that are insensitive to catabolite repression are urgently needed.

The CymR/CuO system of *Pseudomonas putida* F1 (Eaton 1997) consists of the repressor protein CymR and the operator sequence CuO (5′‐AACAAACAGACAATCTGGTCTGTTTGTA‐3′). Upon binding with cumate (4‐isopropylbenzoic acid), CymR undergoes a conformational change and dissociates from CuO, allowing gene expression to proceed. Developing cumate‐inducible promoters in other species has typically involved embedding CuO into constitutive promoters to create hybrid inducible promoters, along with optimised *cymR* expression from constitutively active promoters. Artificially designed cumate‐inducible promoters have been successfully implemented in diverse hosts, including *Alphaproteobacteria* (Kaczmarczyk et al. 2013), *Bacillus* (Seo and Schmidt‐Dannert 2019), *Escherichia coli* (Choi et al. 2010), *Streptomyces* (Horbal et al. 2014), and mammalian cells (Mullick et al. 2006). However, a cumate‐inducible promoter in yeast, particularly in industrially relevant species such as *K. pastoris*, has not yet been reported. Given that cumate is non‐toxic, inexpensive, and not subject to glucose or glycerol repression, it represents a highly attractive inducer for large‐scale bioprocesses.

In this study, we developed a novel cumate‐inducible promoter for *K. pastoris* based on the CymR/CuO regulatory mechanism. By systematically optimising the insertion site of CuO within the strong constitutive promoter P_*GCW14*_ (Liang et al. 2013), we identified several high‐performance hybrid promoters responsive to cumate. We fine‐tuned the expression of CymR through truncation of the strong constitutive promoter P_*GAP*_. The resulting cumate‐inducible promoter P_*gc*_ exhibited high inducibility in *K. pastoris*, showing great promise for this industrially important yeast in metabolic engineering and synthetic biology applications.

## 2 Experimental Procedures

### 2.1 Strains and Media

The *E. coli* Top10 strain was employed for DNA cloning and plasmid propagation, and *K. pastoris* GS115 was used as a host for gene cloning and functional characterisation of the cumate‐inducible promoter. The *E. coli* Top10 strain was routinely cultured in lysogeny broth medium. For cultivation and transformant selection of *K. pastoris* strains, we used yeast peptone dextrose (YPD) medium containing 20 g/L tryptone, 10 g/L yeast extract, and 20 g/L glucose (pH 6.0) and minimal dextrose (MD) medium containing 300 mM potassium phosphate buffer, 13.4 g/L yeast nitrogen base, 4 × 10^−5^% (*w*/*v*) biotin, and 20 g/L glucose. Cumate was added to the media as needed.

### 2.2 Plasmid Construction

A DNA fragment carrying the kanamycin resistance cassette (KanR) and plasmid replicon was amplified from plasmid pEC‐XK99E. Left and right homologous arms for integration into the *gas1* locus of the *K. pastoris* chromosome were cloned from genomic DNA. The *his4* selection marker, used for screening *K. pastoris* transformants, was amplified from plasmid pPIC9. These elements were assembled using a seamless cloning kit to construct the final plasmid, designated as pPICG01.

The promoter P_*GCW14*_ was amplified from *K. pastoris* genomic DNA, and CuO was inserted at various sites within P_*GCW14*_, followed by the T_*act1*_ terminator. These components were then introduced into plasmid pPICG01, yielding a series of plasmids including pPICCuO.

Additionally, either full‐length or truncated P_*GAP*_ was amplified from *K. pastoris* genomic DNA and fused to a codon‐optimised version of the repressor gene *cymR*, which included nuclear localization signals at both ends, along with the T_*cyc1*_ terminator, to form a CymR expression cassette. This cassette was subsequently inserted into pPICCuO to generate the final plasmids, including pPICCymR.

### 2.3 Relative Fluorescence Intensity Assay

The green fluorescent protein (GFP) gene was ligated into the *PmeI* site of the aforementioned plasmids, and the recombinant plasmids thereby generated were introduced into *K. pastoris* by electroporation. The positive transformants were selected on MD medium and then verified by polymerase chain reaction (PCR).

A single positive transformant colony was inoculated into YPD liquid medium and cultured at 30°C with shaking for 48 h. This pre‐culture was transferred at a 1% (v/v) inoculation rate into fresh liquid MD medium with or without cumate and incubated under the same conditions. During the fermentation process, samples were collected at various durations. The GFP fluorescence intensity and optical density at 600 nm (OD_600_) of the cultures were measured using a BioTek Synergy H1 microplate reader (Agilent, Santa Clara, CA, USA). To normalise for cell density variation, relative fluorescence intensity was expressed as the ratio of GFP fluorescence intensity to OD_600_.

All experiments reported in this study were performed in triplicate, and data were presented as the mean ± standard deviation. This same methodology applied to all subsequent experiments described.

### 2.4 Growth Profile of a *K. pastoris* Strain With the *PAS_chr2‐1_0670* Promoter Replaced by the Cumate‐Inducible Promoter P_*gc*_

The left homologous sequence (200–600 bp upstream of the *PAS_chr2‐1_0670* start codon) and the right homologous sequence containing the start codon and an approximately 500‐bp downstream sequence of *PAS_chr2‐1_0670* were amplified from *K. pastoris* chromosomal DNA by PCR. These homologous sequences and the cumate‐inducible promoter were then seamlessly assembled into the gene editing plasmid pPICZAIO via in‐fusion recombination, resulting in a construct designed to replace the native promoter of *PAS_chr2‐1_0670* with the cumate‐inducible promoter. The resulting plasmid was electroporated into competent *K. pastoris* cells, and positive transformants were verified by PCR with the primers 0670‐VF (5′‐CTTATCATCCAGATATCTTTCGGTG‐3′) and 0670‐VR (5′‐GGAGTAGTTAATGGTAATGTCGTGG‐3′). To assess the replacement effect, positive colonies were cultured onto solid YPD medium or in liquid YPD medium with or without 25 mg/L cumate, followed by incubation at 30°C for 48 h. Colony sizes were compared to evaluate phenotypic differences.

## 3 Results

### 3.1 Schematic Diagram of the Cumate‐Inducible System in *K. pastoris*

**FIGURE 1:**
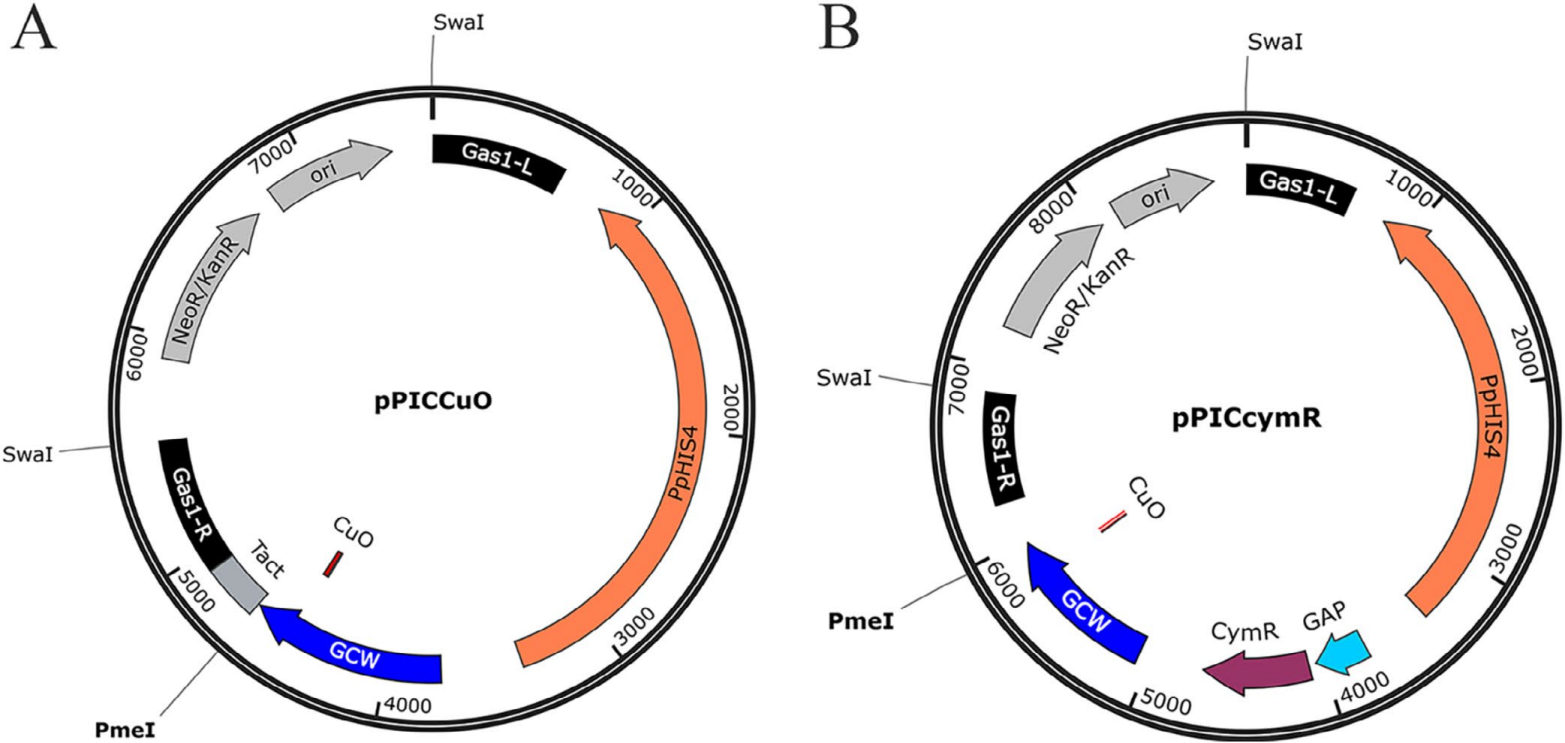
Schematic diagram of the CymR/CuO‐containing plasmids used in this study. (A) pPICCuO and (B) pPICCymR. Gas1‐L and Gas1‐R are homologous sequences for integration at the *gas1* locus; *Pphis4* encodes a multifunctional enzyme required for histidine biosynthesis for transformant selection; GCW is a P_*GCW14*_ modified by the insertion of CuO at different sites; GAP/CymR is a CymR expression cassette in which CymR expression is controlled by the whole or truncated variants of P_*GAP*_.

To develop a strong cumate‐inducible promoter in *K. pastoris*, CuO was inserted at different sites within the strong constitutive promoter P_*GCW14*_ (Liang et al. 2013; Zhang et al. 2013). The modified promoter was subsequently cloned into an integrative plasmid designed for targeted insertion into the *gas1* locus of the *K. pastoris* genome, resulting in plasmids such as pPICCuO (Figure 1A). The GFP gene was placed under the control of these engineered promoters, and their transcriptional activity was assessed by measuring relative GFP fluorescence intensity in the *K. pastoris* transformants.

To evaluate the functionality of the CymR/CuO system in *K. pastoris*, an expression cassette with P_*GAP*_ driving CymR expression was introduced into the plasmid pPICCuO, generating the plasmid pPICCymR (Figure 1B). Then, truncated variants of P_*GAP*_ were constructed and screened to optimise CymR expression. This approach enabled the successful implementation of a tightly regulated, tunable cumate‐inducible gene expression system in *K. pastoris*.

### 3.2 Influence of Cumate on *K. pastoris* Growth

**FIGURE 2:**
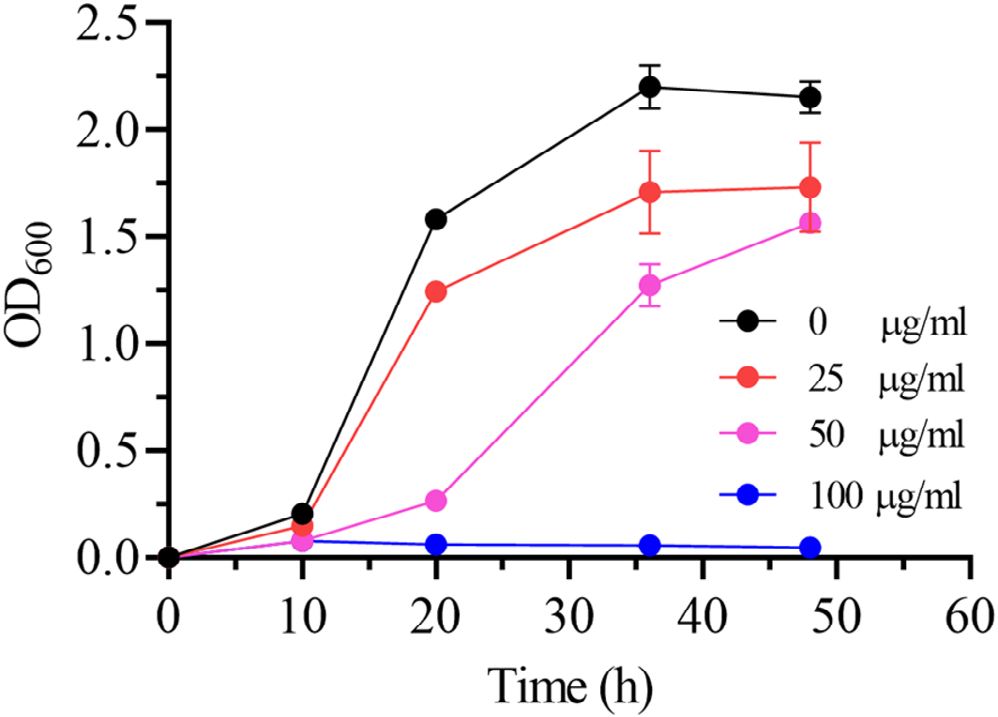
Growth phenotypes of *K. pastoris* GS115 in minimal dextrose (MD) medium containing different concentrations of cumate. Histidine was added to the MD medium to a final concentration of 40 mg/L for strain growth.

Due to the antifungal properties of cumate, it was essential to assess its potential inhibitory effects on *K. pastoris* growth. Inorganic salt media are commonly employed in large‐scale cultivations of *K. pastoris*; therefore, we evaluated the impact of cumate on cell growth under these conditions. The results indicate that 25 mg/L cumate caused only mild inhibition of microbial growth. Although the overall growth trend remained consistent with that of the cumate‐free control, biomass yield was reduced to approximately 78% of the control at comparable time points (Figure 2). When the cumate concentration was increased to 50 mg/L, a significant inhibitory effect was observed, manifesting as a markedly slower growth rate and substantially lower final biomass. Complete growth inhibition occurred at 100 mg/L, with no observable increase in cell density over time (Figure 2). Based on these findings, a concentration of 25 mg/L was selected for subsequent experiments, as it effectively induced gene expression while minimising adverse effects on cell growth.

### 3.3 Effect of CuO Insertion on P_*GCW14*_ Activity

**FIGURE 3:**
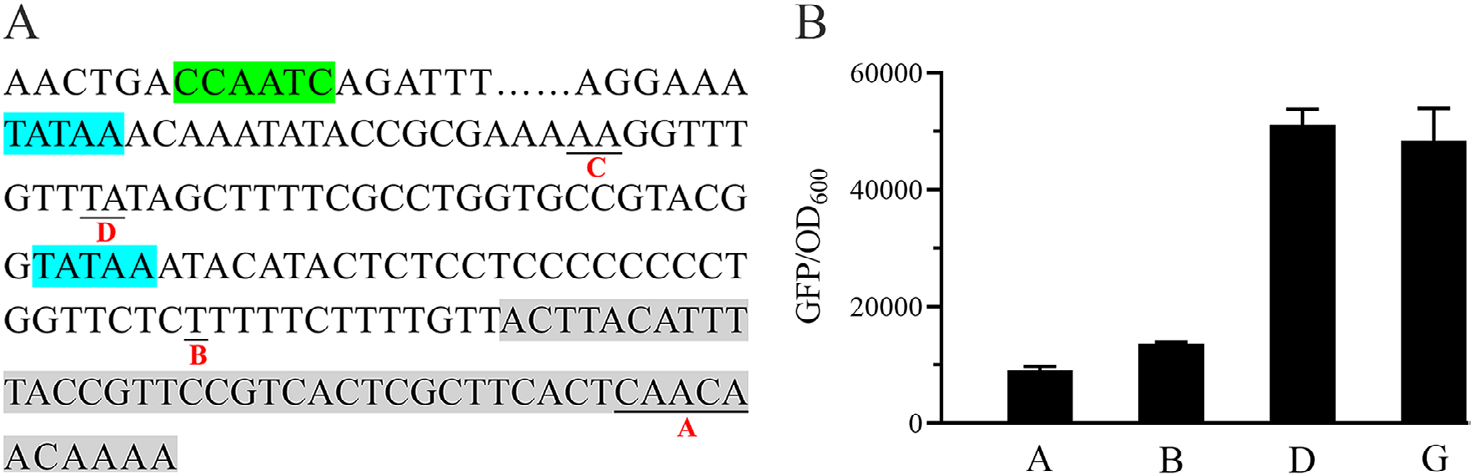
Properties of the modified P_*GCW14*_ with CuO inserted at different sites. (A) Schematic diagram of CuO insertion at different sites. (B) Relative transcriptional activity of different promoters. On the *x*‐axis, A, B and D represent P_*GCW14*_ modified by CuO insertion at sites A, B and D, respectively; G represents the original P_*GCW14*_. Expression of the reporter gene *gfp* was regulated by these promoters; relative transcriptional activity was assessed using green fluorescent protein (GFP) measurements, expressed as relative fluorescence units (RFUs) normalised to the optical density at 600 nm (OD_600_). GFP/OD_600_ was assayed after 48‐h cultivation of the strains in MD medium.

Promoters are critical regulatory elements that determine the expression levels of downstream genes, with strong promoters usually adopted to achieve high‐level gene expression. P_*GCW14*_ has been previously identified as a strong constitutive promoter in *K. pastoris* (Liang et al. 2013; Zhang et al. 2013), making it a suitable candidate for engineering a cumate‐inducible promoter in this species. To develop a cumate‐responsive promoter, CuO was inserted near key transcriptional regulatory elements of P_*GCW14*_: the TATA box and CAAT box (Figure 3A). We assessed the activity levels of the modified promoters using GFP as a reporter gene, with green fluorescence intensity as an indicator of promoter strength.

The results revealed that CuO insertion had a substantial impact on P_*GCW14*_ activity. When CuO was introduced at site A or B, GFP fluorescence intensity decreased by approximately 80% compared to the unmodified P_*GCW14*_. Insertion at site C led to an almost complete loss of fluorescence expression (data not shown). In contrast, when CuO was inserted at site D, fluorescence intensity remained comparable to that of the original promoter, indicating that transcriptional activity was largely preserved (Figure 3B). These findings identified site D as the optimal location for CuO integration. The resulting promoter, P_*GCW14*_ with CuO inserted at site D, was designated as P_*GCWCuO01*_ for subsequent applications.

### 3.4 Effect of CymR Expression Strength on the Inducibility of P_*GCWCuO01*_

It was reported that the degree of repression in the repressible operon of P_lac_/LacI pair was proposed to be primarily determined by the intracellular ratio of repressor molecules to the number of operator sites (Wu et al. 2024), which indicated that the abundance of the repressor protein was a critical determinant of repression efficiency. Similarly, in the CymR/CuO system, CymR binds to CuO of P_*GCWCuO01*_ to inhibit transcription of the downstream gene. The intracellular abundance of CymR is closely linked to both promoter stringency and the amount of inducer used. When CymR is highly abundant, a larger quantity of inducer is required to bind the repressor sufficiently and facilitate its complete dissociation from CuO. In contrast, when CymR levels are low, CuO cannot be fully bound, resulting in significantly leaky expression. Therefore, selecting a promoter with appropriate strength to regulate CymR expression is among the key factors that must be considered in constructing a cumate‐inducible expression system. In this study, another strong promoter from *K. pastoris*, P_*GAP*_, was employed to control CymR expression. We fine‐tuned the expression level of CymR by truncating the promoter, and suitable promoter variants were screened based on the change in GFP fluorescence intensity between uninduced and induced conditions. Of the resulting systems, P_*GCWCuO01*_ controlled *gfp* expression, and the whole P_*GAP*_ promoter or two truncated P_*GAP*_ promoters (P_*GAP50*_ and P_*GAP200*_) regulated CymR expression.

**FIGURE 4:**
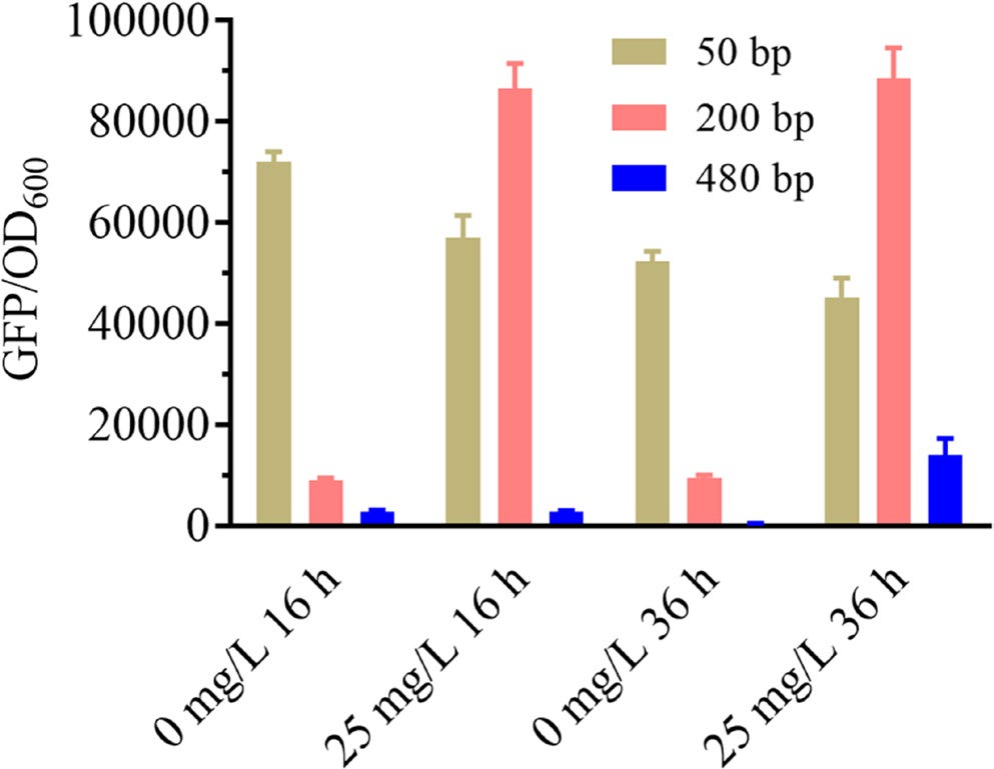
Effect of CymR expression intensity on the induction efficiency of P_*GCWCuO01*_. CymR expression was controlled by truncated variants of P_*GAP*_ (50, 200, and 480 bp). Expression of *gfp* was regulated by P_*GCWCuO01*_ derived from P_*GCW14*_ by inserting one copy of CuO at site D. GFP/OD_600_ was measured after 16‐ and 36‐h cultivation of the strains cultured in MD medium with or without 25 mg/L cumate.

When P_*GAP*_ was truncated to 50 bp, strong green fluorescence was observed both in the absence and presence of the inducer (Figure 4), indicating that the shortened promoter had low transcriptional activity. At this length, it was unable to drive high CymR expression, thus failing to suppress transcription from P_*GCWCuO01*_ effectively. When P_*GAP*_ was truncated to 200 bp, weak fluorescence was detected in the uninduced state, whereas induction led to an approximately nine‐fold increase in green fluorescence intensity (Figure 4). This result implies that CymR was expressed at a moderate level; it effectively bound to CuO and inhibited transcription in the absence of the inducer, while induction successfully relieved this repression. When the full‐length P_*GAP*_ was used to regulate CymR expression, only a weak green fluorescence signal was observed even after induction (Figure 4), although a slight increase was detected. This result implies that CymR was highly expressed but the inducer was insufficient to bind all CymR molecules. As a result, a portion of CymR remained bound to CuO, continuing to inhibit efficient transcription from P_*GCWCuO01*_. Based on these experimental results, the P_*GAP200*_ promoter was identified as being suitable for the regulation of CymR expression, and it was selected for use in subsequent analyses. Accordingly, a cumate‐inducible promoter, P_*gcc01*_, was developed based on P_*GCWCuO01*_ by incorporating a CymR expression unit in which CymR expression was regulated by P_*GAP200*_.

### 3.5 Profiles of P_*GCW14*_ Harbouring Two Copies of CuO

**FIGURE 5:**
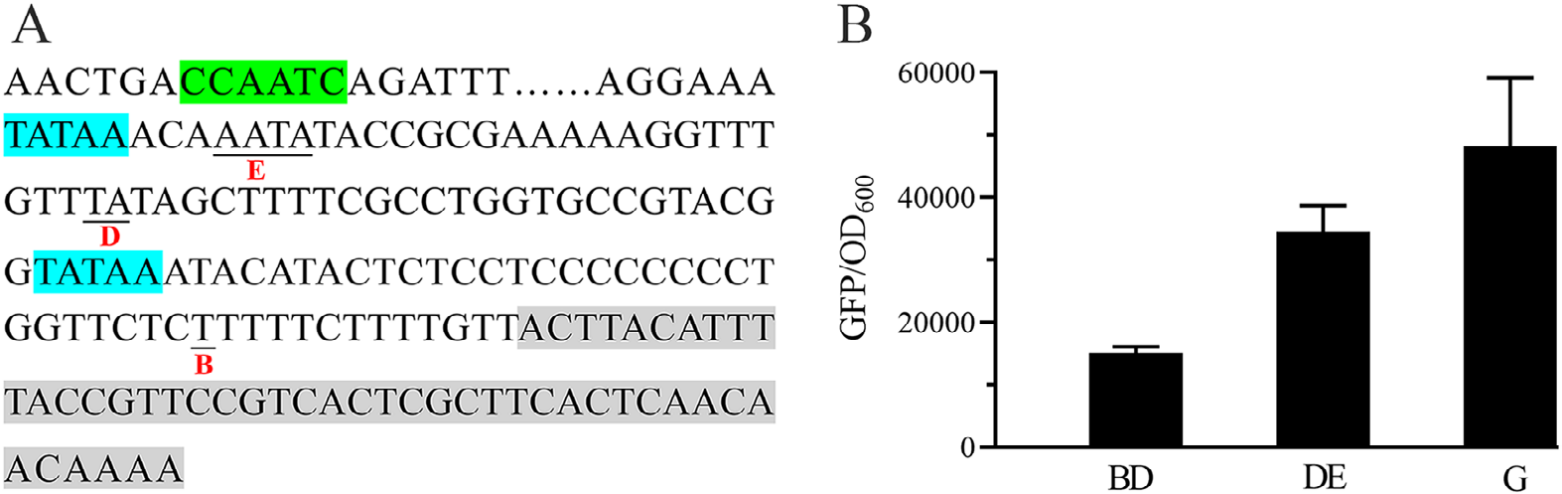
Properties of promoters derived from P_*GCW14*_ by inserting two copies of CuO at two sites. (A) Schematic diagram of CuO inserted at different sites. (B) Relative activity levels of different promoters. The reporter gene *gfp* was regulated by these promoters, and their relative activity was assayed according to GFP/OD_600_. On the *x*‐axis, BD and DE represent P_*GCW14*_ harbouring CuO at sites B and D and sites D and E, respectively; G represents the original P_*GCW14*_. GFP/OD_600_ was measured after 48‐h cultivation of the strains in MD medium.

P_*GCWCuO01*_ harbouring one copy of CuO at site D of P_*GCW14*_ exhibited significantly leaky expression with strong green fluorescence even in the absence of an inducer. To address this issue, an additional CuO was introduced at another site (B or E) of P_*GCWCuO01*_ to develop the promoters P_*GCWCuO02*_ and P_*GCWCuO03*_ (Figure 5A). Our reasoning was that the addition of another CuO sequence might strengthen repression of CymR and reduce the leakiness of the promoter. The transcript profiles of P_*GCWCuO02*_ and P_*GCWCuO03*_ were assayed by determining green fluorescence intensity.

The transcriptional activity of P_*GCWCuO02*_ with CuO insertion at sites B and D resulted in a significant reduction of approximately 70% compared to P_*GCWCuO01*_, indicating that site B was critical for promoter activity. In contrast, promoter P_*GCWCuO03*_ with CuO insertion at sites D and E showed a more modest reduction in transcriptional activity, approximately 30%, compared to P_*GCWCuO01*_ (Figure 5B). Therefore, P_*GCWCuO03*_ was identified as a suitable promoter and selected for further validation.

### 3.6 Profiles of the Cumate‐Inducible Promoter P_*gc*_

Based on P_*GCWCuO03*_ and the CymR expression unit in which CymR expression was regulated by P_*GAP200*_, we developed another novel cumate‐inducible promoter P_*gc*_.

**FIGURE 6:**
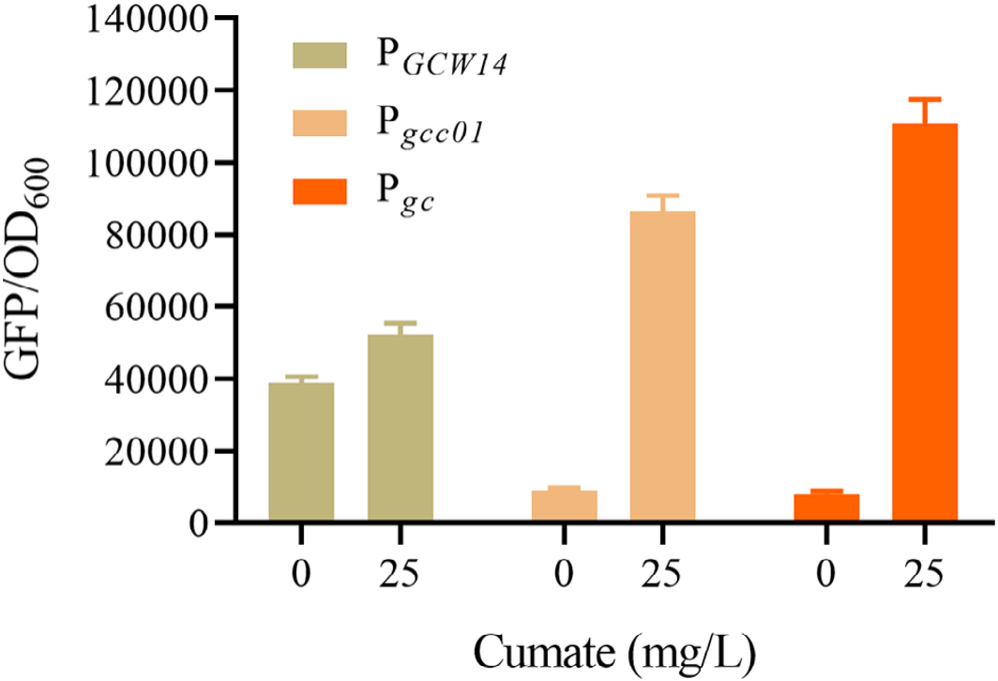
Transcriptional profiles of different promoters. The transcriptional activity, as determined by the GFP/OD_600_ ratio, was measured in strains expressing GFP under the control of various promoters. These strains were cultivated for 48 h in MD medium with or without 25 mg/L cumate. The tested promoters include: P_*GCW14*_, the original P_*GCW14*_; P_*gcc01*_, a modified P_*GCW14*_ with a single CuO copy inserted at site D and CymR expression under the control of P_*GAP*_ with a length of 200 bp; and P_*gc*_, a modified P_*GCW14*_ with two CuO copies inserted at two sites (D and E) and CymR expression under the control of P_*GAP*_ with a length of 200 bp.

The performance of three different promoters, the parental promoter P_*GCW14*_, P_*gcc01*_, and P_*gc*_, was characterised based on GFP fluorescence intensity, respectively. As a constitutive promoter, the transcriptional activity of P_*GCW14*_ did not significantly change in the absence or in presence of cumate (Figure 6). P_*gcc01*_, which contains a single CuO copy in P_*GCW14*_ and a CymR expression unit, was robustly responsive to induction, as indicated by an approximately 8.6‐fold increase in GFP fluorescence from 9000 to 86,000 upon induction with cumate (Figure 6). P_*gc*_, which harbours two copies of CuO in P_*GCW14*_ and a CymR expression unit, showed better inducibility than P_*gcc01*_, as GFP fluorescence increased nearly 13‐fold from 8000 to 110,000 upon induction with cumate (Figure 6). It was surprising that both P_*gcc01*_ and P_*gc*_ exhibited higher transcriptional activities than P_*GCW14*_, which was confirmed through multiple independent experimental replicates. This unexpected result might be attributed to the introduction of the CymR expression unit upstream of the promoter, where sequence‐mediated interactions potentially enhanced the overall transcriptional efficiency. Notedly, P_*gc*_ demonstrated even superior performance to P_*gcc01*_, including an approximate 28% increase in induction strength and an approximate 11% decrease in basal leakage, respectively. The reduction in basal leakage was primarily attributed to the increased number of CuO binding sites, while the precise mechanism underlying this enhancement in transcriptional strength of P_*gc*_ still required further investigation.

### 3.7 Engineering of *K. pastoris* Based on P_*gc*_

**FIGURE 7:**
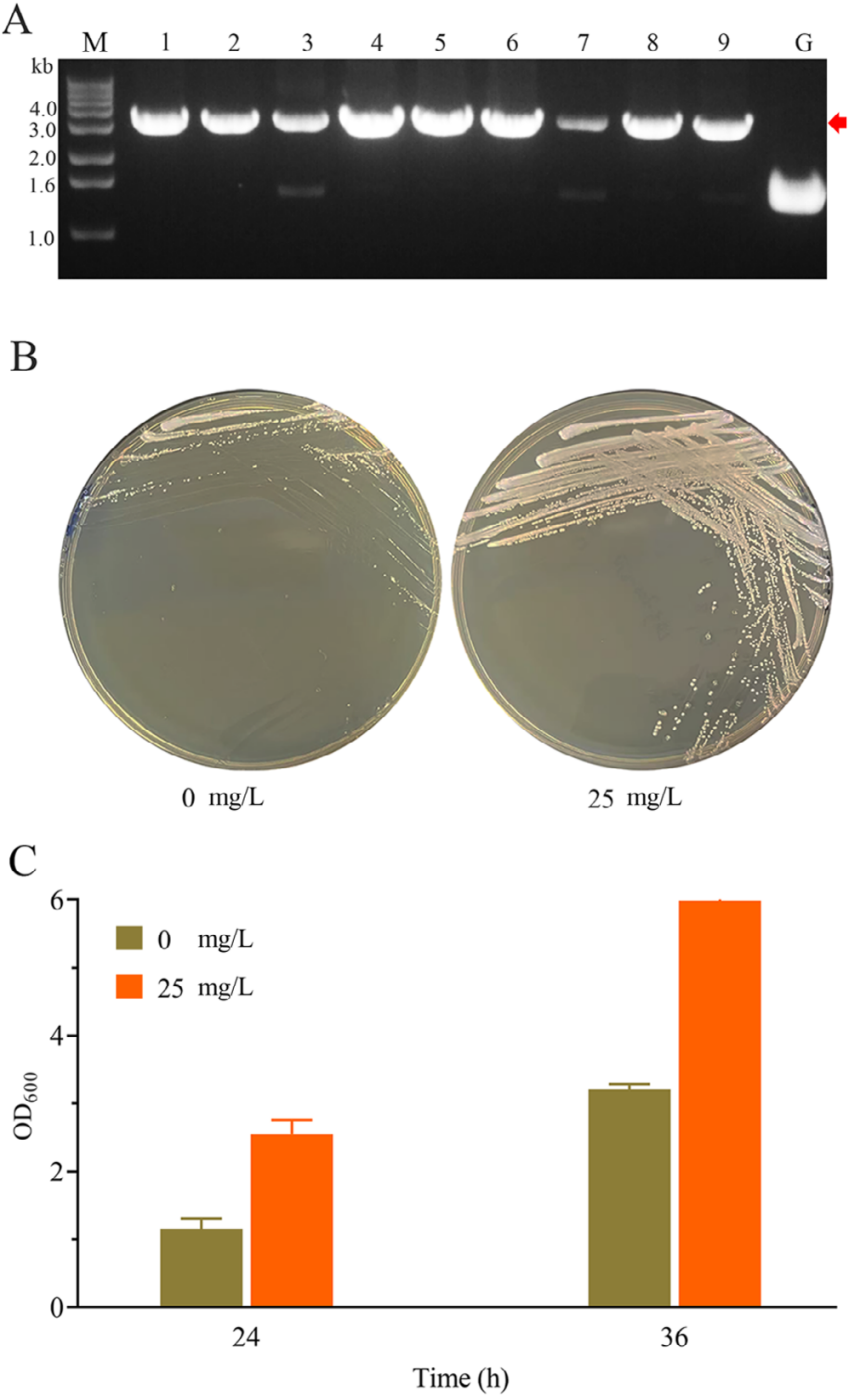
Growth profiles of *K*. *pastoris* GS115C. (A) Verification of *K*. *pastoris* GS115C by polymerase chain reaction (PCR). *K*. *pastoris* GS115C was derived from *K. pastoris* GS115 with *PAS_chr2‐1_0670* promoter replaced by P_*gc*_. The PCR products from *K. pastoris* GS115 and *K. pastoris* GS115C chromosomal DNA were ~1.6 and ~3.5 kb, respectively, with the expected *K. pastoris* GS115C products (~3.5 kb) marked by a red arrow. Lane M, DNA marker; lanes 1–9, PCR products using transformant chromosomal DNA as the templates; lane G, PCR products using *K. pastoris* GS115 chromosomal DNA as the templates. (B) Growth profiles of *K*. *pastoris* GS115C cultured on solid YPD medium without (left) and with (right) cumate of 25 mg/L. (C) Growth profiles of *K. pastoris* GS115C cultured in liquid YPD medium without and with cumate of 25 mg/L.

Our experimental results indicate excellent induction performance by P_*gc*_. To investigate further the effect of this promoter on gene expression regulation in *K. pastoris*, P_*gc*_ was employed for strain engineering. *PAS_chr2‐1_0670* encodes an essential protein localised in the mitochondrial intermembrane space, whose expression level directly influences the growth phenotype of *K. pastoris*. According to previous transcriptomic data (Jiao et al. 2019, 2020), the transcriptional level of *PAS_chr2‐1_0670* is intermediate between that of P_*GCW14*_ and the leaky expression level of P_*gc*_. Therefore, the native promoter of *PAS_chr2‐1_0670* was replaced with P_*gc*_ to achieve more precise regulatory control. The gene editing strategy described by Zhang (Zhang et al. 2021) was adopted to engineer a strain designated as *K. pastoris* GS115C, in which the region 0.2 kb upstream of *PAS_chr2‐1_0670* start codon was replaced with P_*gc*_. The sizes of PCR products amplified from the chromosomal DNA of *K. pastoris* GS115 and the transformants would be ~1.6 and ~3.5 kb, respectively. The expected PCR products were observed (Figure 7A), indicating the successful construction of *K. pastoris* GS115C. Theoretically, in the absence of cumate induction, the transcription level of *PAS_chr2‐1_0670* in *K. pastoris* GS115C should be lower than its original level, leading to a reduced growth rate. Conversely, upon cumate induction, *PAS_chr2‐1_0670* expression was enhanced, thereby restoring growth. Our experimental results aligned well with these predictions; *K. pastoris* GS115C formed smaller colonies on YPD medium without cumate, whereas larger colonies formed on YPD medium supplemented with cumate (Figure 7B). *K. pastoris* GS115C was then cultured in liquid YPD media with and without cumate to quantitatively assess the growth differences. Biomass accumulation was monitored by measuring OD_600_. The results demonstrated that the biomass of *K. pastoris* GS115C grown in cumate‐supplemented media was significantly higher than that in the non‐induced media at equivalent time points (Figure 7C). This finding was consistent with the growth phenotypes observed on solid media, confirming the inducible effect of cumate. These observations implied that the growth rate of *K. pastoris* GS115C was lower in the absence of the inducer and higher under induction, confirming the successful regulation of *PAS_chr2‐1_0670* expression by P_*gc*_.

## 4 Discussion

Promoters are fundamental components of genetic regulation that control the timing, location, and magnitude of gene expression. Inducible promoters are particularly valuable in biotechnology for their capacity to control expression dynamics precisely (Pedone et al. 2019; Tominaga et al. 2024). Therefore, there is increasing interest in developing novel inducible promoter systems that combine high precision, efficiency, and compatibility with industrial‐scale bioprocessing. Several inducible promoters have been established in *K. pastoris*, including the methanol‐inducible P_*AOX1*_, ethanol‐responsive P_*ADH3*_ (Inan and Meagher 2001), and rhamnose‐inducible P_*LRA3*_ (Liu et al. 2016). However, these systems are often subject to carbon catabolite repression in the presence of preferred carbon sources such as glucose or glycerol. Moreover, their practical utility is frequently limited by the nature of the inducers; methanol and ethanol are flammable and pose safety risks, while rhamnose is expensive, collectively increasing both hazards and production costs. Although the copper‐inducible promoter P_*CUP1*_ can be activated by high concentrations of copper ions (Koller et al. 2000), the use of toxic metal ions complicates downstream purification and raises biocompatibility concerns. Thus, there is a clear demand for new promoter systems that are insensitive to carbon catabolite repression and respond to safe, low‐cost, non‐toxic inducers.

In this study, the CymR/CuO system was selected for implementation in *K. pastoris* based on its suitable characteristics. The inducer, cumate, is safe, non‐flammable, non‐toxic, and suitable for industrial fermentation, and the system is not subject to carbon catabolite repression, allowing induction even in repressive carbon sources such as glucose or glycerol. Together, these features enable the combined use of low‐cost carbon sources and cumate, improving process economy and operational safety.

To construct a functional CymR/CuO system, two core elements are required: a promoter containing the operator sequence CuO, and the repressor protein CymR. We selected the strong constitutive promoter P_*GCW14*_ in *K. pastoris* as the backbone for engineering a high‐performance inducible promoter. The CuO operator was inserted into various regions of P_*GCW14*_, which is implicated in transcriptional regulation. Our experimental results indicated that the location of CuO insertion significantly affected promoter activity. Using GFP as a reporter, we constructed a modified version of P_*GCW14*_ with two copies of CuO inserted at an optimal position, designated P_*GCWCuO03*_. This promoter was shown to maintain high transcriptional strength. Control of CymR expression is crucial for system performance; it must be finely tuned to ensure tight repression, minimising basal leakage, without compromising inducibility. To optimise CymR expression, a series of truncated variants of the strong constitutive promoter P_*GAP*_ was constructed. GFP fluorescence assays revealed that shorter (e.g., 50 bp) P_*GAP*_ variants led to insufficient CymR production and elevated basal expression. Through systematic tuning, an optimal CymR expression level was achieved, resulting in a high induction ratio (Figure 6). Unfortunately, a disadvantage of this system was its detectable basal expression although the cumate systems in prokaryotes like *Bacillus* and *E. coli* exhibited tight regulation (Choi et al. 2010; Seo and Schmidt‐Dannert 2019). To reduce leakage further, future designs could incorporate multiple copies of CuO to enhance repressor binding and improve regulatory stringency, as demonstrated in a previous study in which six copies of CuO were inserted into a constitutive promoter (Mullick et al. 2006).

P_*gc*_ represents a powerful, versatile tool for synthetic biology and metabolic engineering in *K. pastoris*. To demonstrate its practical utility, the native promoter of the gene *PAS_chr2‐1_0670* was replaced with P_*gc*_. The resulting transformants exhibited cumate‐dependent growth profiles (Figure 7), illustrating the potential of P_*gc*_ for microbial chassis engineering and functional genomics. This approach provides a robust strategy for constructing tunable cellular systems in which physiological processes can be externally controlled, highlighting its promise for advanced biomanufacturing and synthetic biology applications.

## Author Contributions

J.X. and Y.X.: conducted the experimental investigation and performed the laboratory work. J.X., Y.X., and B.L.: were responsible for writing – original draft, and also contributed to visualization by preparing the figures and tables. H.L., Y.W., W.Z., N.X., and B.L.: participated in writing – review and editing of manuscript drafts and the final version. N.X. and B.L.: contributed to conceptualization, methodology, and research design. All authors were involved in formal analysis, validation, data curation, and results interpretation. Additionally, all authors reviewed and approved the final manuscript.

## Funding

This research was supported by the National Key Research and Development Program of China (2021YFA0910602), the National Natural Science Foundation of China (32472951, 32372921), the China Agriculture Research System of MOF and MARA (CARS‐41), and the Agricultural Science and Technology Innovation Program (CAAS‐ZDRW202304).

## Conflicts of Interest

The authors declare no conflicts of interest.

## Supporting information


**Data S1:** mbt270311‐sup‐0001‐DataS1.docx.

## Data Availability

Data not presented in this article will be made available upon request to the corresponding author.
