# Supplementary material for: Development and Application of a Cumate‐Inducible Promoter, P gc , in Komagataella pastoris
Source: Microb Biotechnol. 2026 Feb 3;19(2):e70311. doi: 10.1111/1751-7915.70311 (PMC12868391; doi:10.1111/1751-7915.70311)
Supplement: Supplementary file 1 — Data S1: mbt270311‐sup‐0001‐DataS1.docx. [file MBT2-19-e70311-s001.docx]

**Development and application of a cumate-inducible promoter,** **P*_gc_*, in *Komagataella pastoris***

Jiachen Xie^1,2#^, Yazhu Xing^3#^, Huiying Luo^3^, Yuan Wang^3^, Wei Zhang^1^, Nan Xu^2^*, Bo Liu^1^*

^1^ Biotechnology Research Institute, Chinese Academy of Agricultural Sciences,

No.12., Zhongguancun South St., Haidian District, Beijing 100081, China.

^2^ College of Forestry, Shanxi Agricultural University,

88 South Daxue Road, Yangzhou 225009, Jiangsu, China

^3^ Institute of Animal Sciences, Chinese Academy of Agricultural Sciences

No. 2 Yuanmingyuan West Road, Haidian, Beijing 100193, China

^#^ Jiachen Xie and Yazhu Xing contributed equally.

Corresponding authors:

Nan Xu

nanxu@yzu.edu.cn

Bo Liu

liubo01@caas.cn

**Sequence of the cumate inducible promoter Pgc in this study**

GTACCCGACCTAGCAGCCCAGGGATGGAAAAGTCCCGGCCGTCGCTGGCAATAATAGCGGGCGGACGCATGTCATGAGATTATTGGAAACCACCAGAATCGAATATAAAAGGCGAACACCTTTCCCAATTTTGGTTTCTCCTGACCCAAAGACTTTAAATTTAATTTATTTGTCCCTATTTCAATCAATTGAACAACTATCAAAACACAATGGGAACTCCAAAGAAGAAAAGAAAGGTCGGAGGTTCTGGTATGAGCCCTAAGCGCCGGACACAAGCGGAGCGTGCCATGGAGACACAGGGGAAGCTAATAGCAGCCGCCCTAGGAGTTCTAAGGGAGAAAGGATACGCGGGATTTCGTATAGCAGATGTACCCGGTGCAGCGGGTGTTTCTAGGGGTGCACAGTCCCACCATTTCCCGACTAAACTGGAACTCCTCTTAGCAACCTTTGAATGGTTATACGAACAAATCACGGAGCGATCGCGAGCTAGGCTGGCTAAGTTGAAACCTGAAGATGACGTCATACAACAGATGTTGGATGATGCAGCCGAATTTTTTCTCGACGACGACTTCTCTATCAGTCTTGACTTGATTGTGGCTGCCGACCGCGATCCGGCGCTTCGGGAGGGCATTCAACGTACTGTTGAGCGTAACCGCTTTGTGGTCGAGGATATGTGGCTTGGCGTGTTGGTTTCGCGGGGACTGAGCCGCGACGACGCTGAAGATATACTCTGGCTGATTTTCAATAGTGTCCGAGGTTTAGCTGTGAGGTCCCTTTGGCAGAAAGATAAGGAGCGATTCGAACGTGTACGGAACTCAACGCTAGAGATCGCCAGAGAACGATATGCGAAATTCAAAAGATCCAGAGCTGACCCAAAGAAGAAGAGAAAAGTATGATCTCTGCTTTTGTGCGCGTGAAGCTTCACGTCCGACGGCGGCCCACGGGTCCCAGGCCTCGGAGATCCGTCCCCCTTTTCCTTTGTCGATATCATGTAATTAGTTATGTCACGCTTACATTCACGCCCTCCCCCCACATCCGCTCTAACCGAAAAGGAAGGAGTTAGACAACCTGAAGTCTAGGTCCCTATTTATTTTTTTATAGTTATGTTAGTATTAAGAACGTTATTTATATTTCAAATTTTTCTTTTTTTTCTGTACAGACGCGTGTACGCATGTAACATTATACTGAAAACCTTGCTTGAGAAGGTTTTGGGACGCTCGAAGGCTTTAATTTGCGTCCAGCCATTTTTTTTCTTTTTTTTTTTTTTATTCAGGTGAACCCACCTAACTATTTTTAACTGGGATCCAGTGAGCTCGCTGGGTGAAAGCCAACCATCTTTTGTTTCGGGGAACCGTGCTCGCCCCGTAAAGTTAATTTTTTTTTCCCGCGCAGCTTTAATCTTTCGGCAGAGAAGGCGTTTTCATCGTAGCGTGGGAACAGAATAATCAGTTCATGTGCTATACAGGCACATGGCAGCAGTCACTATTTTGCTTTTTAACCTTAAAGTCGTTCATCAATCATTAACTGACCAATCAGATTTTTTGCATTTGCCACTTATCTAAAAATACTTTTGTATCTCGCAGATACGTTCAGTGGTTTCCAGGACAACACCCAAAAAAAGGTATCAATGCCACTAGGCAGTCGGTTTTATTTTTGGTCACCCACGCAAAGAAGCACCCACCTCTTTTAGGTTTTAAGTTGTGGGAACAGTAACACCGCCTAGAGCTTCAGGAAAAACCAGTACCTGTGACCGCAATTCACCATGATGCAGAATGTTAATTTAAACGAGTGCCAAATCAAGATTTCAACAGACAAATCAATCGATCCATAGTTACCCATTCCAGCCTTTTCGTCGTCGAGCCTGCTTCATTCCTGCCTCAGGTGCATAACTTTGCATGAAAAGTCCAGATTAGGGCAGATTTTGAGTTTAAAATAGGAAATATAAACA***AACAAACAGACAATCTGGTCTGTTTGTA***TACCGCGAAAAAGGTTTGTT***AACAAACAGACAATCTGGTCTGTTTGTA***TAGCTTTTCGCCTGGTGCCGTACGGTATAAATACATACTCTCCTCCCCCCCCTGGTTCTCTTTTTCTTTTGTTACTTACATTTTACCGTTCCGTCACTCGCTTCACTCAACAACAAAA**GTTTAAAC**TCTCTGCTTTTGTGCGCGTATGTTTATGTATGTACCTCTCTCTCTATTTCTATTTTTAAACCACCCTCTCAATAAAATAAAAATAATAAAGTATTTTTAAGGAAAAGACGTGTTTAAGCACTGACTTTATCTACTTTTTGTACGTTTTCATTGATATAATGTGTTTTGTCTCTCCCTTTTCTACGAAAATTTCAAAAATTGACCAAAAAAAGGAATATATATACGAAAAACTATTATATTTATATATCATAGTGT


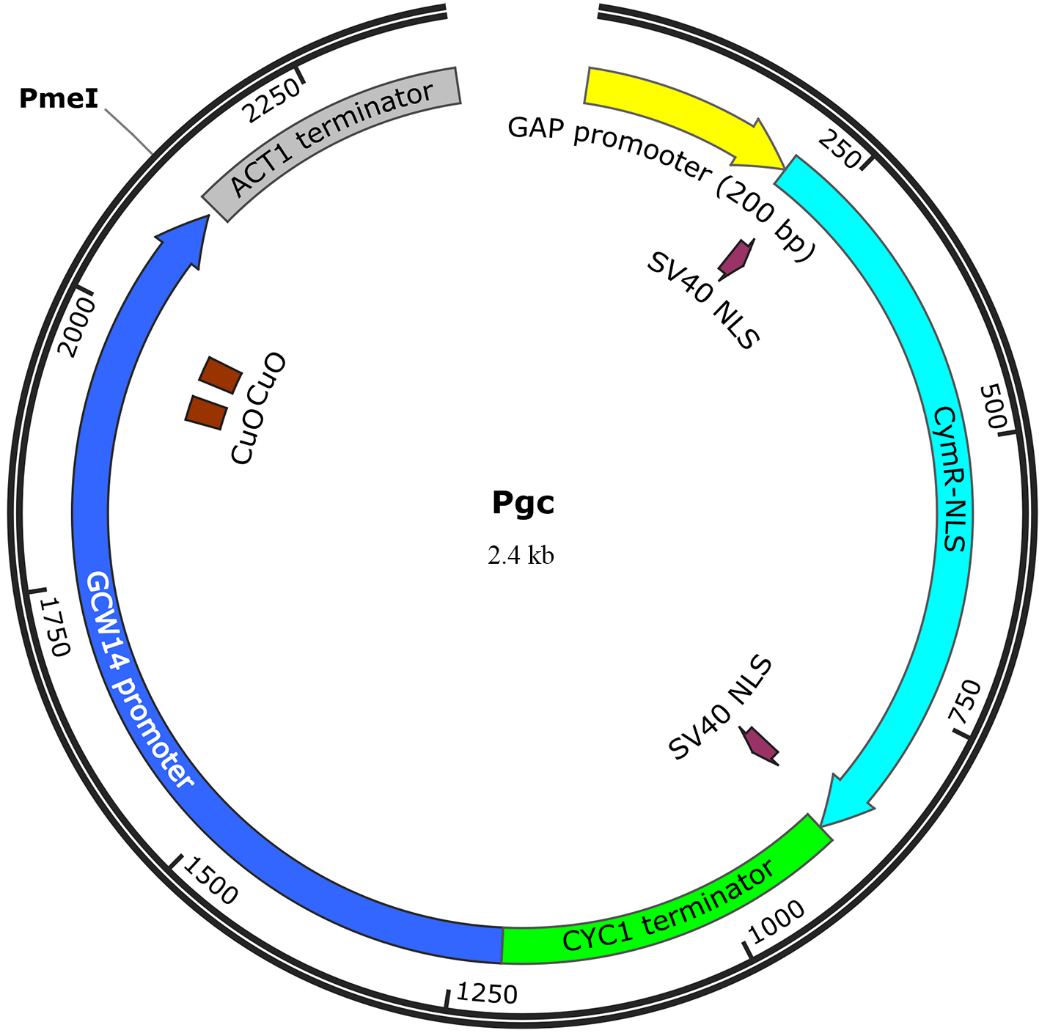


FIGURE S1 Schematic diagram of the cumate inducible promoter in this study. The nuclear localization signal (NLS) and the CuO sequence are underlined and italicized, respectively. The *Pme* I restriction site in the vector can be used for cloning foreign genes.
